# Geographic Variation in Amputations for Medicare Patients With Diabetic Lower-Extremity Wounds

**DOI:** 10.1001/jamanetworkopen.2026.13616

**Published:** 2026-05-19

**Authors:** Ioana Popescu, Helin Hernandez, Gabriela Plasencia, Sarah Schutz, Danya Birnbaum, Jill Gurvey, Aksone Nouvong, Vincent Rowe, Cheryl L. Damberg

**Affiliations:** 1 Division of General Internal Medicine and Health Services Research, David Geffen School of Medicine, University of California, Los Angeles; 2 RAND, Washington, DC; 3 Department of Health Policy, Vanderbilt University, Nashville, Tennessee; 4 RAND, Santa Monica, California; 5 Department of Surgery, Division of Vascular Surgery, David Geffen School of Medicine, University of California, Los Angeles

## Abstract

**Importance:**

Geographic variation in amputations following diabetic lower-extremity (DLE) wounds is substantial. Understanding the sociodemographic and health care factors associated with variations can inform policy to reduce amputations.

**Objective:**

To examine associations of regional patient and health care system characteristics with major amputations following DLE wounds.

**Design, Setting, and Participants:**

This cross-sectional study was conducted among Medicare fee-for-service beneficiaries aged 66 years and older with DLE wounds from 2017 to 2019. Data were aggregated at the hospital referral region (HRR) level. Analyses were performed between February 2025 and February 2026.

**Exposure:**

Diagnosis of a DLE wound.

**Main Outcome and Measures:**

The outcome was the HRR-year proportion of DLE wounds undergoing major amputation within 12 months after diagnosis. Key measures included HRR-year percentages of Black and Hispanic Medicare beneficiaries with DLE wounds, an economic deprivation index, clinician supply per 10 000 Medicare beneficiaries (primary care, endocrinology, podiatry, and revascularization-performing specialists), and the percentage of hospitals with wound management programs. Spatial-temporal bayesian models estimated associations of HRR-year amputations with these measures and were reported as odds ratios (ORs) with 95% credible intervals (CrIs).

**Results:**

The study cohort included 707 971 Medicare DLE wounds (median [range] age, 76 [65-11]; median [range] sex, 55.0% [44.0%-68.0%] male) in 306 HRRs with wide variation in median (range) racial composition (5.2% [0.0%-53.1% Black and 1.4% [0.0%-87.0%] Hispanic), economic deprivation index (60.6 [7.2-87.8]), specialist supply per 10 000 Medicare beneficiaries (podiatry: 4.0 [1.1-16.3]; endocrinology: 1.4 [0.2-5.4]; revascularization-performing specialties: 8.0 [3.9-28.0]) and the percentage of hospitals with wound management programs (63.4% [25.0%-100%]). The median (range) HRR-year proportion of DLE wounds followed by major amputation was 3.0% (0.3%-9.3%). In bayesian models, the percentages of Black (OR, 5.19; 95% CrI, 3.78-7.12) and Hispanic patients (OR, 2.39; 95% CrI, 1.73-3.29) and economic deprivation (OR, 1.29; 95% CrI, 1.02-1.62) had positive associations with amputations. Among health care factors, only HRR-level podiatrist supply had a negative association with amputations (OR, 0.74; 95% CrI, 0.66-0.82), corresponding to an estimated 19.1%, 6.6%, and 3.2% reduction in amputation odds with 1 additional podiatrist per 10 000 Medicare beneficiaries in low-, medium-, and higher-supply markets (approximately 1, 4, and 9 podiatrists per 10 000 beneficiaries).

**Conclusions and Relevance:**

In this cross-sectional study of Medicare beneficiaries with DLE wounds, beyond social determinants of health, higher podiatrist supply was associated with fewer amputations. These findings suggest that increasing access to podiatry may reduce amputations among patients with DLE wounds.

## Key Points

**Question:**

What factors are associated with geographic variations in amputations for patients with diabetic lower-extremity (DLE) wounds?

**Findings:**

In this cross-sectional study of 707 971 Medicare DLE wounds in 306 hospital referral regions, higher regional-level economic deprivation and proportions of Black and Hispanic patients were associated with higher proportions of major amputations following DLE wounds. Among health care factors, higher podiatrist supply was associated with substantially fewer amputations, while other specialist supply and the market-level proportion of hospitals with wound management programs were not associated with amputations.

**Meaning:**

These findings suggest that beyond addressing social determinants of health, regional interventions to increase podiatry supply or compensate for lack of podiatry-specific expertise may decrease DLE wound-related amputations.

## Teaser

This cross-sectional study examines associations of regional patient and health care system characteristics with major amputation following a diabetic lower-extremity wound among Medicare beneficiaries.

## Introduction

Diabetic lower-extremity (DLE) wounds are among the most serious complications of diabetes, affecting up to 34% of patients with diabetes during their lifetimes.^1^ Approximately 1 in 5 DLE wounds leads to amputation,^2^ resulting in significant clinical and economic consequences for patients and health care systems alike.^2^

The burden of DLE wounds and related amputations is unevenly distributed across geographies and demographic groups. Studies reveal substantial variations across census regions, health care markets, and metropolitan areas,^3,4,5^ suggesting that local health system factors may play an influential role in the management of DLE wounds. Persistent disparities also exist among racial, ethnic, and socioeconomic lines; American Indian and Alaska Native, Black, Hispanic, and low-income populations experience the highest rates of DLE wound-related amputations.^4,6,7^ These patterns likely reflect a combination of patient-, social-, and system-level factors—including disparities in health literacy, diabetic foot care education and preventive services,^8,9,10^ delays in seeking care,^11,12^ limited access to specialized or multidisciplinary services,^12,13^ local shortages in health care resources,^13^ and variation in quality of care.^12^

Although amputation risk following DLE wounds arises from complex pathways, health system factors are increasingly recognized as modifiable targets for intervention. Evidence shows that timely specialty care—especially multidisciplinary approaches—can prevent major amputations and reduce hospitalizations and mortality among patients with diabetic foot ulcers.^14,15^ Hospital-based wound care programs, with their ability to centralize expertise and leverage advanced technologies,^16^ may be particularly efficient at improving wound healing and reducing adverse outcomes for patients with DLE wounds.^17^ However, the effectiveness of multidisciplinary wound management programs, whether hospital- or community-based, also depends on their specialty composition,^18^ which is subject to local specialist (eg, podiatrist, vascular surgeon, and endocrinologist) supply shortages.^19,20,21^

The objective of this study was to examine how regional health care system characteristics—particularly specialist supply and the availability of hospital-based wound management programs—and the sociodemographic composition of the regional patient population are associated with variation in major amputations after DLE wound diagnosis. We selected Dartmouth Atlas hospital referral regions (HRRs)^22^ as the unit of analysis because they represent regional markets for complex tertiary care and align with our focus on regional DLE wound management capacity. Because wound management programs are often organized in affiliation with large hospitals and tertiary centers serving multiple catchment areas, HRRs capture the broader local resources available to patients with DLE wounds. The study population comprised older Medicare beneficiaries, a group with particularly high prevalence of diabetes and diabetes-related complications.^23^

## Methods

This cross-sectional study followed the Strengthening the Reporting of Observational Studies in Epidemiology (STROBE) reporting guideline. The study was approved by the RAND institutional review board, and a waiver of informed consent was granted because of its retrospective nature. Analyses were performed between February 2025 and February 2026. The study team used Medicare claims and enrollment data that were obtained under a data use agreement with the Centers for Medicare & Medicaid Services (CMS). Researchers interested in accessing the Medicare data need to apply for and obtain approval for data access and enter into a data use agreement with CMS.

### Data Sources and Study Population

We used 2016 to 2020 100% Medicare inpatient, outpatient, carrier (physician), skilled nursing facility, and home health agency claims for fee-for-service (FFS) beneficiaries aged 66 years and older diagnosed with a DLE wound between 2017 and 2019 and their health care utilization claims from 2016 to 2020. The study cohort consisted of patients with DLE wound episodes diagnosed from 2017 to 2019 who were followed up for 12 months after DLE wound diagnosis for the occurrence of major (above-ankle) amputations. We defined DLE wounds using* International Statistical Classification of Diseases, Tenth Revision, Clinical Modification (ICD-10-CM)* codes for lower-extremity wounds and diabetes (eTable 1 in Supplement 1) and adapting previously published methods.^24^ Additionally, we excluded wounds with an associated *ICD-10-CM* diagnostic code suggestive of venous stasis ulcer at the time of diagnosis (eTable 1 in Supplement 1). To ensure completeness of data, we excluded patients without continuous Medicare part A and B enrollment.

We used the American Hospital Association (AHA) annual survey data for 2016 to 2020 to ascertain the availability of hospital-based wound management programs. To ensure the appropriate denominator for hospital-based wound management capacity for FFS Medicare beneficiaries, we restricted the analysis to privately owned or nonfederal government hospitals (excluding federal hospitals such as Veterans Affairs Hospitals) and to general medical surgical, rehabilitation, or long-term acute care hospitals (ie, those likely to treat chronic wounds).

### Key and Control Variables

The unit of analysis was the Dartmouth Atlas HRR; HRRs cover the 50 US states and the District of Columbia. All study variables were measured at the HRR level per study year. The study outcome was the percentage of DLE wounds followed by major amputations. Key exposure variables included sociodemographic HRR characteristics, the presence of hospital-based wound management programs, and relevant specialist supply.

#### Major Amputation

Major amputation after a DLE wound was identified using specific *Current Procedural Terminology* codes present on claims for 12 months after diagnosis (eTable 1 in Supplement 1) and aggregated at the HRR-year level. We excluded traumatic amputations, which were identified based on specific *ICD-10-CM* diagnostic codes S88.xx occurring in association with amputation *Current Procedural Terminology* codes. All amputations were attributed to the year the DLE wound was diagnosed (eg, if a wound was diagnosed in 2017 but the amputation occurred in 2018 then the amputation was attributed to 2017).

#### Hospital-Based Wound Management Program Capacity

We measured HRR hospital-based wound management capacity as the proportion of hospitals with programs among all HRR hospitals per study year. We ascertained whether study hospitals (5061 hospitals) had a wound management program in each study year using their responses to the annual AHA survey. The AHA survey includes a single item on wound management, with response options indicating whether the hospital has an on-site wound management program and whether the hospital accesses wound services through system affiliations or other contractual arrangements (yes, no, or missing for each). For this study, we classified hospitals based only on reports of an on-site wound management program, consistent with prior work demonstrating benefits of these programs.^16,25^ Among study hospitals, 582 (11.5%) missed reporting this indicator variable in 1 year, 439 (8.7%) missed reporting this indicator in 2 years, and 886 (17.5%) missed this variable for all 3 study years. We imputed missing data using 2016 to 2020 AHA survey responses. For the main analysis, we applied a last observation carried forward approach, imputing a wound management program as present when it was reported in the prior year but missing in the current year. In sensitivity analyses, we used a mirrored next observation carried backward approach. Details are provided in the eMethods in Supplement 1.

#### Clinician Supply

We defined supply as the number of specialists treating patients with DLE wounds per 10 000 Medicare FFS beneficiaries in the HRR per study year, representing the realized supply available to Medicare beneficiaries in the market. We obtained the total number of FFS beneficiaries from Medicare enrollment files. We used Medicare 2-digit specialty codes on claims to identify treating physicians in relevant specialties including primary care, endocrinology, podiatry, vascular surgery, interventional cardiology, interventional radiology, and other relevant surgical specialties (ie, general and cardiac surgery). Because of their overlapping role in wound management, we summed the supply of revascularization-performing specialists (vascular surgeons, cardiologists, and interventional radiologists) per HRR; in sensitivity analyses, we also modeled these supply variables separately. To construct HRR-level measures, we assigned clinicians to HRRs based on the residence of the patients with DLE wounds they treated. If clinicians treated patients from multiple HRRs, we apportioned them across HRRs according to the share of their Medicare DLE wound claims in each region (eg, 30% of claims in HRR 1 and 70% in HRR 2 contributed 0.3 and 0.7 clinician equivalents to those HRRs). Because the association of clinician supply with outcomes is not linear, we log-transformed supply measures in our models.

#### Key and Control HRR Demographic, Economic and Clinical Characteristics

We measured 3 HRR-level demographic and economic characteristics per study year: proportions of Black and Hispanic Medicare patients with DLE wounds, economic deprivation, and rurality. Race and ethnicity were ascertained using information from the Medicare base enrollment files and a bayesian probability algorithm,^26^ which employs additional beneficiary information (surname, Census Block Group) to improve race and ethnicity identification from enrollment files as is widely applied in Medicare data analyses.^27,28,29^ Although prior research has identified 3 high-risk groups (American Indian and Alaska Native, Black, and Hispanic), we included only the proportions of Black and Hispanic patients because the American Indian and Alaska Native population was small and highly clustered in a few HRRs, which would have compromised the reliability and stability of HRR-level estimates. We constructed an HRR-level economic deprivation index by aggregating zip code level Area Deprivation Index (ADI) values from the 2015 Neighborhood Atlas,^30^ weighted by zip code population size from the US Census.^31^ Rurality was measured using rural-urban commuting area codes^32^ to classify residential zip codes as urban or rural, and HRR rurality was defined as the proportion of the population living in rural zip codes, following a similar approach as for the ADI index.

Demographic and clinical control variables included age, sex, Medicaid dual eligibility (a marker of individual low-income^33^), coexisting peripheral arterial disease (PAD) and a diabetes complications and severity index (DCSI).^34^ Age, sex, and dual eligibility were obtained from Medicare enrollment files; patients were classified as dual-eligible if enrolled for at least 1 month in a study year. Coexisting PAD (eTable 1 in Supplement 1) and the DCSI (eTable 2 in Supplement 1) were identified using *ICD-10-CM* diagnosis codes from claims in the 12 months before DLE wound diagnosis, adapting published methods.^34^ All covariates were first defined at the wound level and then aggregated at the HRR level per study year using means and proportions.

### Statistical Analysis

We examined variations in HRR characteristics using descriptive statistics (range, mean, median, and SD) and heat maps to visualize geographic patterns for key variables. We then used a bayesian hierarchical logistic regression model to estimate associations of HRR-level exposures (percentages of Black and Hispanic patients with DLE wounds, rurality, economic deprivation index, specialist supply, and the proportion of hospitals with wound management programs) with the likelihood of major amputations after DLE wound diagnosis. Models adjusted for HRR-level aggregates of patient covariates (mean age, mean DCSI score, and the percentages of male patients, dual-eligible patients, and patients with PAD) and included spatial and temporal random effects to capture residual similarities in practice patterns between neighboring HRRs as well as changes in practice patterns over time. Model results are presented as odds ratios (ORs) and 95% credible intervals (CrIs), which are interpreted similarly to confidence intervals (ie, we considered an association to be statistically important when the 95% CrI for the odds ratio excluded the null value of 1); we also used bayesian posterior probabilities of 97.5 or greater and 2.5 or less to assess the magnitude of the observed association as large-positive and large-negative, respectively. All analyses were performed using SAS 9.4 (SAS Institute) and R version 4.5.2 (R Project for Statistical Computing) and the INLA package version 25.10.19.

## Results

### Descriptive Findings

The study sample included 707 971 Medicare DLE wounds in 306 HRRs (median [range] age, 76 [65-11]; median [range] sex, 55.0% [44.0%-68.0%] male). Table 1 shows pooled summaries of HRR characteristics across the 3-year study period. Key characteristics varied considerably across HRRs, including the median (range) proportion of Medicare DLE wounds resulting in amputations (3.0% [0.3%-9.3%]), median (range) proportion of Black (5.2% [0.0%-53.1%]) and Hispanic (1.4% [0.0%-86.9%]) Medicare patients with DLE wounds, median (range) economic deprivation index (60.5 [7.2-87.8]), median (range) proportion of population in rural zip codes (19.8% [0.0%-100.0%]), median (range) proportion of hospitals with wound management programs (63.4% [25.0%-100.0%]), and key specialist supply per 10 000 Medicare beneficiaries (podiatry: 4.0 [1.1-16.3]; revascularization-performing specialists: 8.0 [3.9-28.0]; endocrinology: 1.4 [0.2-5.4]).

**Table 1.** HRR Characteristics Across the Study Period (2017-2019)

HRR-level characteristics	Median (range)
DLE wounds undergoing amputation at 12 mos, %	3.0 (0.3-9.3)
Medicare patients with a DLE wound by race and ethnicity, %	
Black	5.2 (0.0-53.1)
Hispanic	1.4 (0.0-86.9)
Economic deprivation, population-weighted ADI	60.5 (7.2-87.8)
Rural population, %	19.8 (0.0-100.0)
Study hospitals, No.	11.2 (2.0-92.3)
Hospitals with wound management programs, %	63.4 (25.0-100.0)
Primary care physician supply^a^	52.9 (28.2-128.5)
Podiatrist supply^a^	4.0 (1.1-16.3)
Revascularization-performing specialist supply^a^	8.0 (3.9-28.0)
Endocrinologist supply^a^	1.4 (0.2-5.4)
Other relevant surgical specialty supply^a^	7.0 (3.4-14.3)

Abbreviations: ADI, Area Deprivation Index; DLE, diabetic lower extremity; HRR, hospital referral region.

^a^ Measured as number of specialists for every 10 000 fee-for-service Medicare beneficiaries.

**Figure 1.**
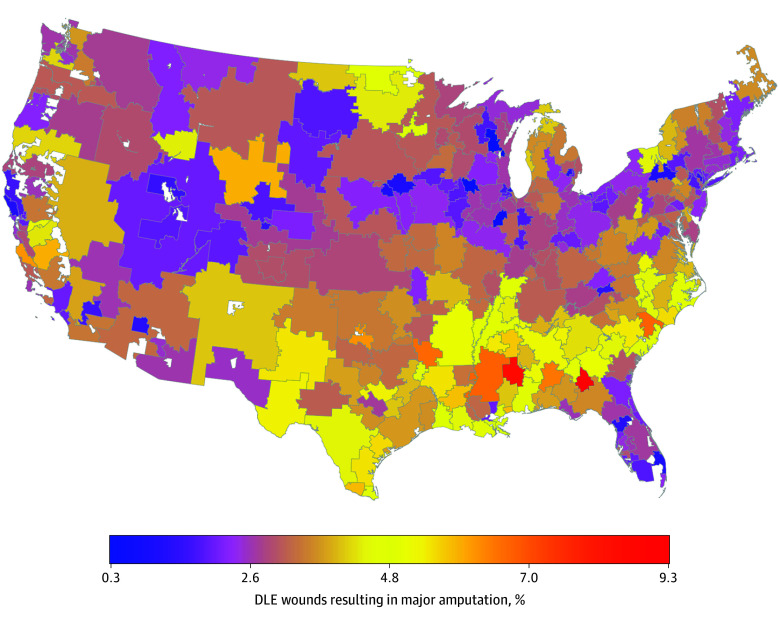
Heat Map of the Mean Percentage of Diabetic Lower-Extremity (DLE) Wounds Resulting in Major Amputation, 2017-2019 Alaska and Hawaii are omitted from the map for visualization purposes but were included in the model. The percentages of major amputation after wound diagnosis for these 2 hospital referral regions were 3.5% and 2.8%, respectively.

**Figure 2.**
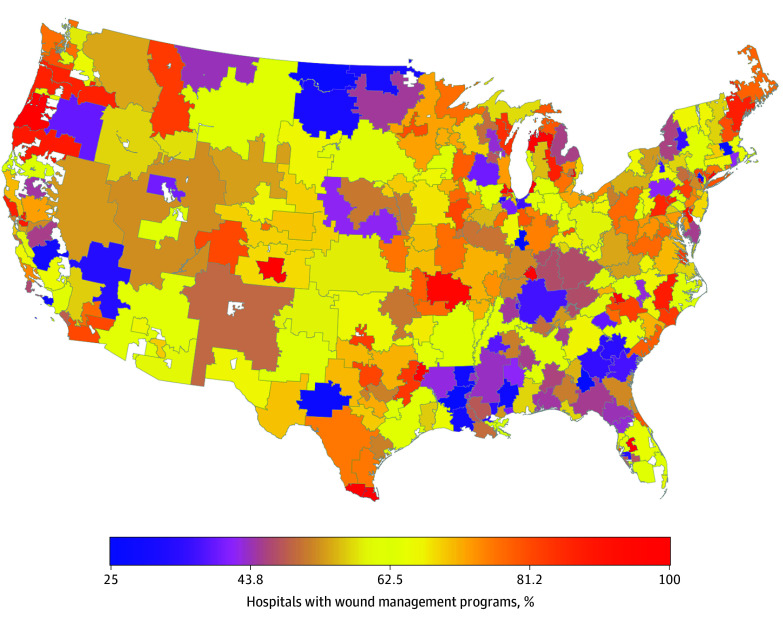
Heat Map of the Mean Percentage of Hospitals With Wound Management Programs, 2017-2019 Alaska and Hawaii are omitted from the map for visualization purposes but were included in the model. The percentage of hospitals with wound management programs in these 2 hospital referral regions were 46.0% and 58.0%, respectively.

**Figure 3.**
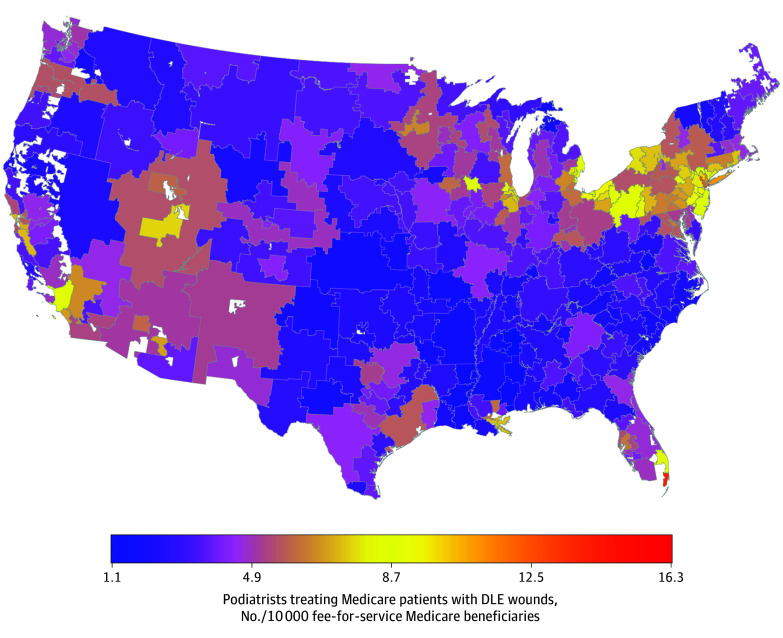
Heat Map of the Mean Number of Podiatrists Treating Medicare Patients With Diabetic Lower-Extremity (DLE) Wounds per 10 000 Fee-For-Service Medicare Beneficiaries, 2017-2019 Alaska and Hawaii are omitted from the map for visualization purposes but were included in the model. The podiatrist supply for these 2 hospital referral regions was 3.4 and 3.5 per 10 000 fee-for-service Medicare beneficiaries, respectively.

Heat maps further illustrate geographic variations in these key variables. The proportion of DLE wounds followed by major amputation was generally higher in South and Southwest, with a few hot spots in other HRRs (Figure 1). The proportion of hospitals with wound management programs also varied widely within states and regions (Figure 2). The supply of podiatrists appeared generally low and concentrated in a few HRRs located in the Northeast and select urban areas (New York City, Chicago, Miami, and Los Angeles) (Figure 3). Substantial geographic variations in the proportion of Black and Hispanic patients with DLE wounds, economic deprivation, and rurality are presented in eFigures 1 to 4 in Supplement 1.

### Model Results

Table 2 presents the associations of amputations after DLE wound diagnosis with key market characteristics. As discussed, model results are represented as bayesian posterior probabilities, ORs, and 95% CrIs, along with the combined interpretation of these statistics. The percentage of Black (OR, 5.19; 95% CrI, 3.78-7.12) and Hispanic patients (OR, 2.39; 95% CrI, 1.73-3.29) and the population-weighted ADI index (OR, 1.29; 95% CrI, 1.02-1.62) showed large-magnitude positive associations with the proportion of amputations 12 months after wound diagnosis. Among specialist supply factors, only podiatrist supply had a large negative association with amputations (OR, 0.74; 95% CrI, 0.66-0.82). Using model coefficients, we then estimated that by adding 1 podiatrist per 10 000 Medicare beneficiaries, amputation odds would decrease by 19.1% in HRRs with 1 podiatrist per 10 000 beneficiaries, 6.6% in HRRs with 4 podiatrists per 10 000 beneficiaries, and 3.2% in HRRs with 9 podiatrists per 10 000 Medicare beneficiaries, respectively.

**Table 2.** Adjusted Associations of Market-Level Characteristics and Amputations for Medicare Beneficiaries Diagnosed With Diabetic Lower-Extremity Wounds, 2017-2019

HRR variable	OR (95% CrI)^a^
Percentage of Black Medicare patients	5.19 (3.78-7.12)^b^
Percentage of Hispanic Medicare patients	2.39 (1.73-3.29)^b^
Population-weighted economic deprivation index (ADI)	1.29 (1.02-1.62)^b^
Rural population percentage	1.12 (0.94-1.35)
Percentage of hospitals with wound management programs	1.02 (0.87-1.18)
Primary care supply	1.19 (0.98-1.45)
Podiatry supply	0.74 (0.66-0.82)^c^
Revascularization-performing specialist supply	1.03 (0.87-1.21)
Endocrinology supply	0.99 (0.92-1.07)
Other relevant surgical specialties	1.06 (0.90-1.24)

Abbreviations: ADI, Area Deprivation Index; CrI, credible interval; OR, odds ratio.

^a^ The 95% CrI is interpreted similarly to a confidence interval, in that there is 95% probability that the true OR lies within the credible interval range.

^b^ The bayesian posterior probabilities are 97.5% or greater, indicating a large-magnitude positive association.

^c^ The bayesian posterior probability is 2.5% or less, indicating a large-magnitude negative association.

Other key variables, including HRR rurality and the proportion of hospitals with wound management programs, were not associated with amputations (Table 2). Sensitivity analyses using the alternative imputation algorithm for missing hospital-based wound management program information (eTable 3 in Supplement 1), and those modeling the 3 revascularization-performing specialties supply variables, separately showed similar results.

## Discussion

As diabetes and its complications become increasingly prevalent, identifying regional factors that may contribute to or prevent poor outcomes is essential for improving the health and quality of life of people with diabetes. In this cross-sectional study of US Medicare beneficiaries with DLE wounds treated within regional health care markets, we found that market-level racial and ethnic composition and economic deprivation were important risk factors for amputation rates. Among health system factors, higher market-level podiatrist supply was associated with fewer amputations, whereas other specialist supply and hospital-based wound management capacity were not associated with amputations. The finding that race and ethnicity and area-level economic deprivation had large-magnitude associations with higher amputation rates after DLE wound diagnosis is well established in the literature on diabetes-related adverse outcomes.^35,36^ Further, higher proportions of Black and Hispanic patients with DLE wounds at the market level may indicate a concentration of social and structural risk factors such as differential access to preventive care and timely specialty services, disparities in health literacy, and financial and transportation barriers, that could contribute to higher amputation rates in those markets. Our results highlight the public health implications of these associations; in markets with high concentrations of such social risk factors, targeted interventions are needed to reduce preventable amputations. These might include health system–community partnerships to improve diabetes and foot-care quality, tailored health education on wound prevention and early symptom recognition, expanded access to podiatry and vascular evaluation (eg, through outreach clinics or telehealth), and social support programs to address, nutrition, transportation, caregiving, and other barriers.

The most important finding from our analysis is that greater podiatrist supply was associated with fewer amputations. In low-supply markets, each additional podiatrist per 10 000 Medicare beneficiaries was associated with an almost 20% reduction in amputation odds for patients with DLE wounds, underscoring the central role of podiatry in DLE wound care. Podiatrists bring direct wound care expertise (pressure offloading, debridement, advanced dressings, and specialized footwear prescriptions^37^) and may provide key preventive services to high-risk patients with diabetes (eg, those with kidney failure, PAD, or advanced neuropathy) that lower the risk of major amputation.^38^ Finally, podiatrists may serve as critical connectors between primary care physicians, specialists, and ancillary services participating in DLE referral networks, a hypothesis that deserves further investigation.

Despite clear benefits, the US faces ongoing and projected podiatrist shortages.^39^ While uncapped Medicare funding has doubled residency positions between 1998 and 2018, workforce projections still estimate a shortfall exceeding 4000 podiatrists by 2030,^40^ which corresponds to roughly 20% of the currently practicing podiatry workforce according to the American Podiatric Medical Association. The residency program expansion alone does not ensure improved supply, as applicant numbers to podiatric schools are declining.^41^ Increasing student recruitment will be crucial to addressing shortages. Federal policy also does not address the maldistribution and particularly severe shortages of podiatrists in rural and economically deprived areas. Targeted interventions, adapted from those addressing shortages in other clinical expertise —such as training more advanced practice clinicians (eg, nurse practitioners) who can manage select wound care tasks, or providing financial incentives—may also help.

Contrary to expectations, a higher proportion of hospitals with wound management programs in the market was not associated with lower amputation rates at 1 year. While longer-term benefits are still possible, future longitudinal studies are needed to clarify this relationship. The lack of a clear association may also reflect heterogeneity in the types of services offered by wound management programs, referral team structure, and care continuity within the program, which can affect program effectiveness.^16,25,42^ Notably, multidisciplinary wound management programs, although potentially beneficial, can be difficult for health systems to sustain—especially in resource-constrained settings—due to marginal cost-effectiveness and organizational challenges.^25^ More granular, longitudinal primary data collection is likely needed to characterize variation in wound program size, structure, and specific services offered, and to assess their association with amputation prevention.

### Limitations

The study has several limitations. First, analyses are cross-sectional and aggregated at the regional health care market level; thus, it is difficult to infer causal relationships between market characteristics and amputation rates. Second, the variable representing the proportion of Black and Hispanic beneficiaries with DLE wounds reflects both the underlying racial and ethnic composition of the HRR and any disproportionate burden among Black and Hispanic patients; given the ecologic design, we cannot disentangle these mechanisms. Third, the wound management capacity measure was constructed from hospital-level data with substantial missingness, which we addressed using multiple imputation at the hospital level; as a result, the measure may misclassify the presence, intensity, or quality of multidisciplinary limb-salvage efforts. However, by averaging the measure of hospital-based wound management capacity across many hospitals per region, we reduced its sensitivity to any single imputed value.

## Conclusions

In this market-level cross-sectional study of Medicare beneficiaries with DLE wounds, we found key associations of regional sociodemographic characteristics and podiatry supply with amputation rates after wound diagnosis. To our knowledge, this is the first national, population-based study to highlight the association of podiatry supply with amputations among patients with DLE wounds. While sociodemographic factors are difficult to address, optimizing the current workforce, strengthening DLE wound referral networks, and expanding access to podiatric expertise may deliver more immediate improvements as broader, long-term policy solutions are being developed.

## Notes

Supplement 1. eTable 1. ICD-10 Diagnostic Codes Used to Identify DLE Wounds and Preexisting PAD eMethods. Imputation Algorithm for Missing AHA Survey Information on Hospital-Based Wound Management Programs eTable 2. Diabetes Complication Severity Index (DCSI) Components and Weights eFigure 1. Percentage of Black DLE Wound Medicare Patients, Averaged for 2017-2019 eFigure 2. Percentage of Hispanic DLE wound Medicare patients, Averaged for 2017-2019 eFigure 3. Population-Weighted Area Deprivation Index (ADI), Averaged for 2017-2019 eFigure 4. Population-Weighted Rurality, Averaged for 2017-2019 eTable 3. Bayesian Multivariable Model Results: Comparing Main Analysis and Sensitivity Analysis Results Using Alternative Imputation Methods for Missing AHA Survey Information on Hospital-Based Wound Management Programs

Supplement 2. Data Sharing Statement
