# Supplementary material for: Geographic Variation in Amputations for Medicare Patients With Diabetic Lower-Extremity Wounds
Source: JAMA Netw Open. 2026 May 19;9(5):e2613616. doi: 10.1001/jamanetworkopen.2026.13616 (PMC13187873; doi:10.1001/jamanetworkopen.2026.13616)
Supplement: Supplement 1. — eTable 1. ICD-10 Diagnostic Codes Used to Identify DLE Wounds and Preexisting PAD eMethods. Imputation Algorithm for Missing AHA Survey Information on Hospital-Based Wound Management Programs eTable 2. Diabetes Complication Severity Index (DCSI) Components and Weights eFigure 1. Percentage of Black DLE Wound Medicare Patients, Averaged for 2017-2019 eFigure 2. Percentage of Hispanic DLE wound Medicare patients, Averaged for 2017-2019 eFigure 3. Population-Weighted Area Deprivation Index (ADI), Averaged for 2017-2019 eFigure 4. Population-Weighted Rurality, Averaged for 2017-2019 eTable 3. Bayesian Multivariable Model Results: Comparing Main Analysis and Sensitivity Analysis Results Using Alternative Imputation Methods for Missing AHA Survey Information on Hospital-Based Wound Management Programs [file jamanetwopen-e2613616-s001.pdf]

Supplemental Online Content

Popescu I, Hernandez H, Placensia G. Geographic variation in amputations for Medicare patients with diabetic lower extremity wounds. *JAMA Netw. Open*. 2026;9(5):e2613616. doi:10.1001/jamanetworkopen.2026.13616

eTable 1. *ICD-10* Diagnostic Codes Used to Identify DLE Wounds and Preexisting PAD
eMethods. Imputation Algorithm for Missing AHA Survey Information on Hospital-Based Wound Management Programs
eTable 2. Diabetes Complication Severity Index (DCSI) Components and Weights
eFigure 1. Percentage of Black DLE Wound Medicare Patients, Averaged for 2017-2019
eFigure 2. Percentage of Hispanic DLE wound Medicare patients, Averaged for 2017-2019
eFigure 3. Population-Weighted Area Deprivation Index (ADI), Averaged for 2017-2019
eFigure 4. Population-Weighted Rurality, Averaged for 2017-2019
eTable 3. Bayesian Multivariable Model Results: Comparing Main Analysis and Sensitivity Analysis Results Using Alternative Imputation Methods for Missing AHA Survey Information on Hospital-Based Wound Management Programs

This supplemental material has been provided by the authors to give readers additional information about their work.

© 2026 Popescu I et al. *JAMA Network Open*.

eTable 1. ***ICD-10*** **diagnostic codes used to identify DLE wounds and preexisting PAD**

**Condition**	**Corresponding ICD 10 diagnostic codes or CPT codes**	**Number and type of claims to qualify**
LE wound* ICD-10	L97.201, L97.202, L97.203, L97.204, L97.205, L97.206, L97.208, L97.209, L97.211, L97.212, L97.213, L97.214, L97.215, L97.216, L97.218, L97.219, L97.221, L97.222, L97.223, L97.224, L97.225, L97.226, L97.228, L97.229, L97.301, L97.302, L97.303, L97.304, L97.305, L97.306, L97.308, L97.309, L97.311, L97.312, L97.313, L97.314, L97.315, L97.316, L97.318, L97.319, L97.321, L97.322, L97.323, L97.324, L97.325, L97.326, L97.328, L97.329, L97.401, L97.402, L97.403, L97.404, L97.405, L97.406, L97.408, L97.409, L97.411, L97.412, L97.413, L97.414, L97.415, L97.416, L97.418, L97.419, L97.421, L97.422, L97.423, L97.424, L97.425, L97.426, L97.428, L97.429, L97.501, L97.502, L97.503, L97.504, L97.505, L97.506, L97.508, L97.509, L97.511, L97.512, L97.513, L97.514, L97.515, L97.516, L97.518, L97.519, L97.521, L97.522, L97.523, L97.524, L97.525, L97.526, L97.528, L97.529, L97.801, L97.802, L97.803, L97.804, L97.805, L97.806, L97.808, L97.809, L97.811, L97.812, L97.813, L97.814, L97.815, L97.816, L97.818, L97.819, L97.821, L97.822, L97.823, L97.824, L97.825, L97.826, L97.828, L97.829, L97.901, L97.902, L97.903, L97.904, L97.905, L97.906, L97.908, L97.909, L97.911, L97.912, L97.913, L97.914, L97.915, L97.916, L97.918, L97.919, L97.921, L97.922, L97.923, L97.924, L97.925, L97.926, L97.928, L97.929<br>(any diagnosis on the claim)	At least one claim on Inpatient, Outpatient, Carrier or SNF files during 2017-2019
Diabetes ICD-10	E08.00, E08.01, E08.10, E08.11, E08.21, E08.22, E08.29, E08.311, E08.319, E08.321, E08.3211, E08.3212, E08.3213, E08.3219,E08.329, E08.3291, E08.3292, E08.3293, E08.3299, E08.331, E08.3311, E08.3312, E08.3313, E08.3319, E08.339, E08.3391, E08.3392, E08.3393, E08.3399, E08.341, E08.3411, E08.3412, E08.3413, E08.3419, E08.349, E08.3491, E08.3492, E08.3493, E08.3499, E08.351, E08.3511, E08.3512, E08.3513, E08.3519, E08.3521, E08.3522, E08.3523, E08.3529, E08.3531, E08.3532, E08.3533, E08.3539, E08.3541, E08.3542, E08.3543, E08.3549, E08.3551, E08.3552, E08.3553, E08.3559, E08.359, E08.3591, E08.3592, E08.3593, E08.3599, E08.36, E08.37X1, E08.37X2, E08.37X3, E08.37X9, E08.39, E08.40, E08.41, E08.42, E08.43, E08.44, E08.49, E08.51, E08.52, E08.59, E08.610, E08.618, E08.620, E08.621, E08.622, E08.628, E08.630, E08.638, E08.641, E08.649, E08.65, E08.69, E08.8, E08.9, E09.00, E09.01,	At least one claim on Inpatient or SNF files or 2 claims on Outpatient or Carrier files during 12 months prior to wound diagnosis

© 2026 Popescu I et al. *JAMA Network Open*.

	E09.10, E09.11, E09.21, E09.22, E09.29, E09.311, E09.319, E09.321, E09.3211, E09.3212, E09.3213, E09.3219, E09.329, E09.3291, E09.3292, E09.3293, E09.3299, E09.331, E09.3311, E09.3312, E09.3313, E09.3319, E09.339, E09.3391, E09.3392, E09.3393, E09.3399, E09.341, E09.3411, E09.3412, E09.3413, E09.3419, E09.349, E09.3491, E09.3492, E09.3493, E09.3499, E09.351, E09.3511, E09.3512, E09.3513, E09.3519, E09.3521, E09.3522, E09.3523, E09.3529, E09.3531, E09.3532, E09.3533, E09.3539, E09.3541, E09.3542, E09.3543, E09.3549, E09.3551, E09.3552, E09.3553, E09.3559, E09.359, E09.3591, E09.3592, E09.3593, E09.3599, E09.36, E09.37X1, E09.37X2, E09.37X3, E09.37X9, E09.39, E09.40, E09.41, E09.42, E09.43, E09.44, E09.49, E09.51, E09.52, E09.59, E09.610, E09.618, E09.620, E09.621, E09.622, E09.628, E09.630, E09.638, E09.641, E09.649, E09.65, E09.69, E09.8, E09.9, E10.10, E10.11, E10.21, E10.22, E10.29, E10.311, E10.319, E10.321, E10.3211, E10.3212, E10.3213, E10.3219, E10.329, E10.3291, E10.3292, E10.3293, E10.3299, E10.331, E10.3311, E10.3312, E10.3313, E10.3319, E10.339, E10.3391, E10.3392, E10.3393, E10.3399, E10.341, E10.3411, E10.3412, E10.3413, E10.3419, E10.349, E10.3491, E10.3492, E10.3493, E10.3499, E10.351, E10.3511, E10.3512, E10.3513, E10.3519, E10.3521, E10.3522, E10.3523, E10.3529, E10.3531, E10.3532, E10.3533, E10.3539, E10.3541, E10.3542, E10.3543, E10.3549, E10.3551, E10.3552, E10.3553, E10.3559, E10.359, E10.3591, E10.3592, E10.3593, E10.3599, E10.36, E10.37X1, E10.37X2, E10.37X3, E10.37X9, E10.39, E10.40, E10.41, E10.42, E10.43, E10.44, E10.49, E10.51, E10.52, E10.59, E10.610, E10.618, E10.620, E10.621, E10.622, E10.628, E10.630, E10.638, E10.641, E10.649, E10.65, E10.69, E10.8, E10.9, E11.00, E11.01, E11.10, E11.11, E11.21, E11.22, E11.29, E11.311, E11.319, E11.321, E11.3211, E11.3212, E11.3213, E11.3219, E11.329, E11.3291, E11.3292, E11.3293, E11.3299, E11.331, E11.3311, E11.3312, E11.3313, E11.3319, E11.339, E11.3391, E11.3392, E11.3393, E11.3399, E11.341, E11.3411, E11.3412, E11.3413, E11.3419, E11.349, E11.3491, E11.3492, E11.3493, E11.3499, E11.351, E11.3511, E11.3512, E11.3513, E11.3519, E11.3521, E11.3522, E11.3523, E11.3529, E11.3531, E11.3532, E11.3533, E11.3539, E11.3541, E11.3542, E11.3543, E11.3549, E11.3551, E11.3552, E11.3553, E11.3559, E11.359, E11.3591, E11.3592, E11.3593, E11.3599, E11.36, E11.37X1, E11.37X2, E11.37X3, E11.37X9, E11.39, E11.40, E11.41, E11.42, E11.43,	

© 2026 Popescu I et al. *JAMA Network Open*.

	E11.44, E11.49, E11.51, E11.52, E11.59, E11.610, E11.618, E11.620, E11.621, E11.622, E11.628, E11.630, E11.638, E11.641, E11.649, E11.65, E11.69, E11.8, E11.9, E13.00, E13.01, E13.10, E13.11, E13.21, E13.22, E13.29, E13.311, E13.319, E13.321, E13.3211, E13.3212, E13.3213, E13.3219, E13.329, E13.3291, E13.3292, E13.3293, E13.3299, E13.331, E13.3311, E13.3312, E13.3313, E13.3319, E13.339, E13.3391, E13.3392, E13.3393, E13.3399, E13.341, E13.3411, E13.3412, E13.3413, E13.3419, E13.349, E13.3491, E13.3492, E13.3493, E13.3499, E13.351, E13.3511, E13.3512, E13.3513, E13.3519, E13.3521, E13.3522, E13.3523, E13.3529, E13.3531, E13.3532, E13.3533, E13.3539, E13.3541, E13.3542, E13.3543, E13.3549, E13.3551, E13.3552, E13.3553, E13.3559, E13.359, E13.3591, E13.3592, E13.3593, E13.3599, E13.36, E13.39, E13.40, E13.41, E13.42, E13.43, E13.44, E13.49, E13.51, E13.52, E13.59, E13.610, E13.618, E13.620, E13.621, E13.622, E13.628, E13.630, E13.638, E13.641, E13.649, E13.65, E13.69, E13.8, E13.9<br>(any diagnosis on the claim)	
PAD ICD-10	I700, I701, I702, I70201, I70202, I70203, I70208, I70209, I70211, I70212, I70213, I70218, I70219, I70221, I70222, I70223, I70228, I70229, I70231, I70232, I70233, I70234, I70235, I70238, I70239, I70241, I70242, I70243, I70244, I70245, I70248, I70249, I7025, I70261, I70262, I70263, I70268, I70269, I70291, I70292, I70293, I70298, I70299, I70301, I70302, I70303, I70308, I70309, I70311, I70312, I70313, I70318, I70319, I70321, I70322, I70323, I70328, I70329, I70331, I70332, I70333, I70334, I70335, I70338, I70339, I70341, I70342, I70343, I70344, I70345, I70348, I70349, I7035, I70361, I70362, I70363, I70368, I70369, I70391, I70392, I70393, I70398, I70399, I70401, I70402, I70403, I70408, I70409, I70411, I70412, I70413, I70418, I70419, I70421, I70422, I70423, I70428, I70429, I70431, I70432, I70433, I70434, I70435, I70438, I70439, I70441, I70442, I70443, I70444, I70445, I70448, I70449, I7045, I70461, I70462, I70463, I70468, I70469, I70491, I70492, I70493, I70498, I70499, I70501, I70502, I70503, I70508, I70509, I70511, I70512, I70513, I70518, I70519, I70521, I70522, I70523, I70528, I70529, I70531, I70532, I70533, I70534, I70535, I70538, I70539, I70541, I70542, I70543, I70544, I70545, I70548, I70549, I7055, I70561, I70562, I70563, I70568, I70569, I70591, I70592, I70593, I70598, I70599, I70601, I70602, I70603, I70608, I70609, I70611, I70612, I70613, I70618, I70619, I70621, I70622, I70623, I70628, I70629, I70631, I70632, I70633, I70634, I70635, I70638, I70639, I70641, I70642,	At least one claim on Inpatient or SNF files or 2 claims on Outpatient or Carrier files during 12 months prior to initial DLE wound diagnosis

© 2026 Popescu I et al. *JAMA Network Open*.

	I70643, I70644, I70645, I70648, I70649, I7065, I70661, I70662, I70663, I70668, I70669, I70691, I70692, I70693, I70698, I70699, I70701, I70702, I70703, I70708, I70709, I70711, I70712, I70713, I70718, I70719, I70721, I70722, I70723, I70728, I70729, I70731,I70732, I70733, I70734, I70735, I70738, I70739, I70741, I70742, I70743, I70744, I70745, I70748, I70749, I7075, I70761, I70762, I70763, I70768, I70769, I70791, I70792, I70793, I70798, I70799, I708, I7090, I7091, I7092, I7100, I7101, I7102, I7103, I711, I712, I713, I714, I715, I716, I718, I719, I720, I721, I722, I723, I724, I725, I726, I728, I729, I7300, I7301, I731, I7381, I7389, I739, I7401, I7409, I7410, I7411, I7419, I742, I743, I744, I745, I748, I749, I75011, I75012, I75013, I75019, I75021, I75022, I75023, I75029, I7581, I7589, I770, I771, I772, I775, I7770, I7771, I7772, I7773, I7774, I7775, I7776, I7777, I7779, I77810, I77811, I77812, I77819, I7789, I779, I780, I781, I788, I789, I790, I791, I798 (any diagnosis on the claim)	
Venous stasis with ulcer ICD 10	I83012, I83013, I83022, I83023, I8320, I8321, I8322, I87.311, I87.312, I87.313, I87.319	At least one claim on Inpatient, Outpatient, Carrier or SNF files at the time of DLE wound diagnosis
Above knee CPT	27590, 27591, 27592, 27594, 27596, 27598	Specific code present on Carrier or Outpatient claim on the day of or within 12 months following the initial DLE wound diagnosis AND no ICD10 diagnostic code of "S88.X" on any claim on +/-2 days of amputation procedure date.
Above ankle/ below knee CPT	27880, 27881, 27882, 27884, 27886, 27888, 27889	

*We excluded L97.1 (non-pressure chronic ulcer of thigh) as it is an unlikely DLE wound event

© 2026 Popescu I et al. *JAMA Network Open*.

eTable 2. Diabetes Complication Severity Index (DCSI) components* and weights

COMPLICATION	CODES	SCORE
**EYE**		
**Severe**		
Proliferative retinopathy	E08.35, E09.35, E10.35, E11.35, E13.35	2
Retinal detachment	H33	2
Vitreous hemorrhage	H43.1	2
Blindness	H54	2
**Moderate**		
Non-proliferative retinopathy	E08.31, E09.31, E10.31, E11.31, E13.31, E08.32, E09.32, E10.32, E11.32, E13.32, E08.33, E09.33, E10.33, E11.33, E13.33, E08.34, E09.34, E10.34, E11.34, E13.34, E08.37, E09.37, E10.37, E11.37, E13.37	1
**RENAL**		
Severe	E08.2, E09.2, E10.2, E11.2, E13.2 AND any of: N18.4, N18.5, N18.6, Z99.2	2
Moderate	E08.2, E09.2, E10.2, E11.2, E13.2 AND any of: N18.1, N18.2, N18.3, N19	1
NEUROLOGICAL		
Peripheral neurological complications	E08.4, E09.4 E10.4, E11.4, E13.4	1
Cerebrovascular		
TIA (if no CVA)	G45	1
CVA	I63	2
Coronary heart disease		
non-AMI	I24, I25	1
AMI	I21	2
METABOLIC		
Ketoacidosis	E08.1, E09.1, E10.1, E11.1, E13.1	2
Hyperosmolarity	E08.0, E09.0, E10.0, E11.0, E13.0	2

*The original DCSI score includes PAD; given we measured PAD as an independent risk factor, it was not included in the score. TIA= Transient Ischemic Attack; CVA = Cerebrovascular Accident; AMI= Acute Myocardial Infarction.

© 2026 Popescu I et al. *JAMA Network Open*.

eMethods

Imputation algorithm for missing AHA survey information on hospital-based wound management programs

The AHA survey possible responses to the question of whether a hospital had a wound management program were 1 (yes) 0 (no), or missing. Among 5061 study hospitals, 582 (11.5%) had a missed value for answer to the AHA question in one year, 439 (8.7%) missed reporting this indicator in 2 years, and 886 (17.5%) missed this variable for all three study years. We used information reported by these hospitals on the 2016-2020 AHA surveys to impute missing responses.

The main imputation method used a "last observation carried forward" approach, carrying forward the previous year responses. For single-year imputations, if the previous year response value was 1 and the following year response value was 0 or missing, the missing year value was imputed as 1. If the previous year response value was 0 or missing and the following year response value was 1, the missing year response value was imputed as 0.

For consecutive missing years, the same logic was applied. Specifically, if the prior year response value was 0 or missing and the following year was 1, all consecutive missing years were imputed as 0. If the prior year response value was 1 and the following year response value was 0 or missing, all consecutive missing years were imputed as 1. As an example, for hospitals missing 2017-2018 data, if the 2016 response value was 0 or missing and the 2019 response value was 1, both 2017 and 2018 were imputed as 0. Alternatively, if the 2016 value was 1 and the 2019 value was missing or 0, both 2017 and 2018 were imputed as 1.

Finally, for non-consecutive missing years (i.e., missing 2017 and 2019), the imputation logic was applied independently to each missing year. To evaluate the robustness of this assumption, a sensitivity analysis was conducted using a next observation carried backward, in which missing years were imputed based on the following year's value rather than the prior year's value, therefore reversing the main imputation algorithm.

© 2026 Popescu I et al. *JAMA Network Open*.

eFigure 1. Percentage of Black DLE wound Medicare patients, averaged for 2017-2019

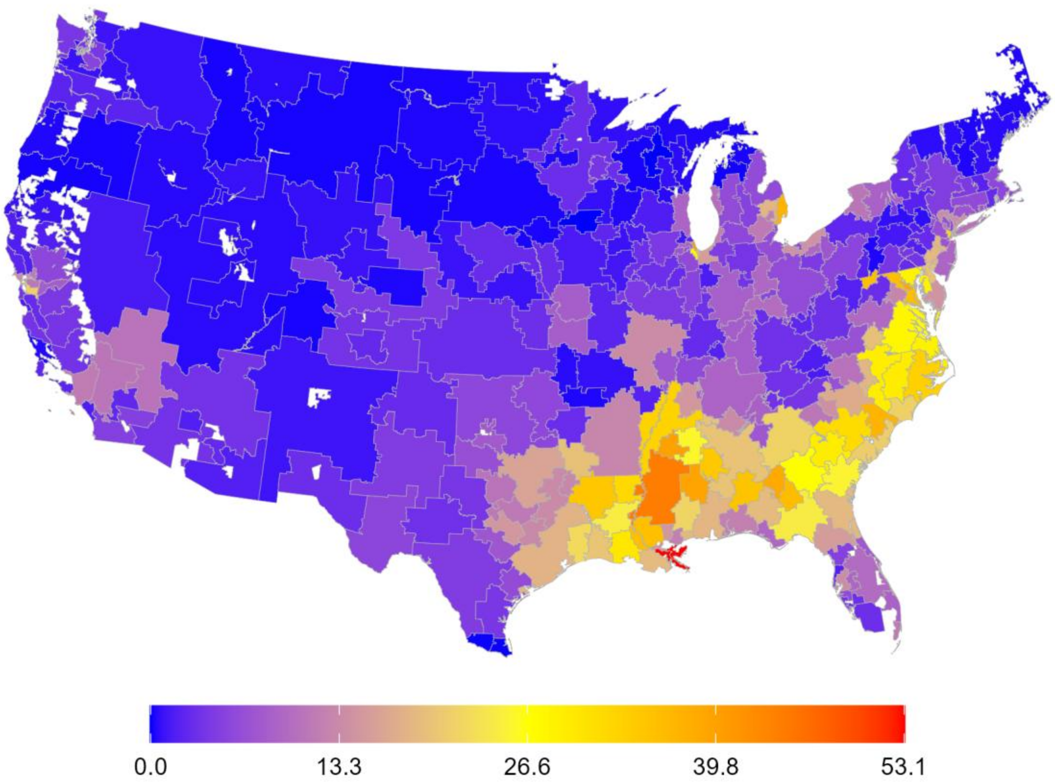

Note: Alaska and Hawaii HRRs are omitted from the map for visualization purposes but are included in the model. The proportions of Black DLE wound patients were 4.3% and 1.1%, respectively.

© 2026 Popescu I et al. *JAMA Network Open*.

eFigure 2. Percentage of Hispanic DLE wound Medicare patients, averaged for 2017-2019

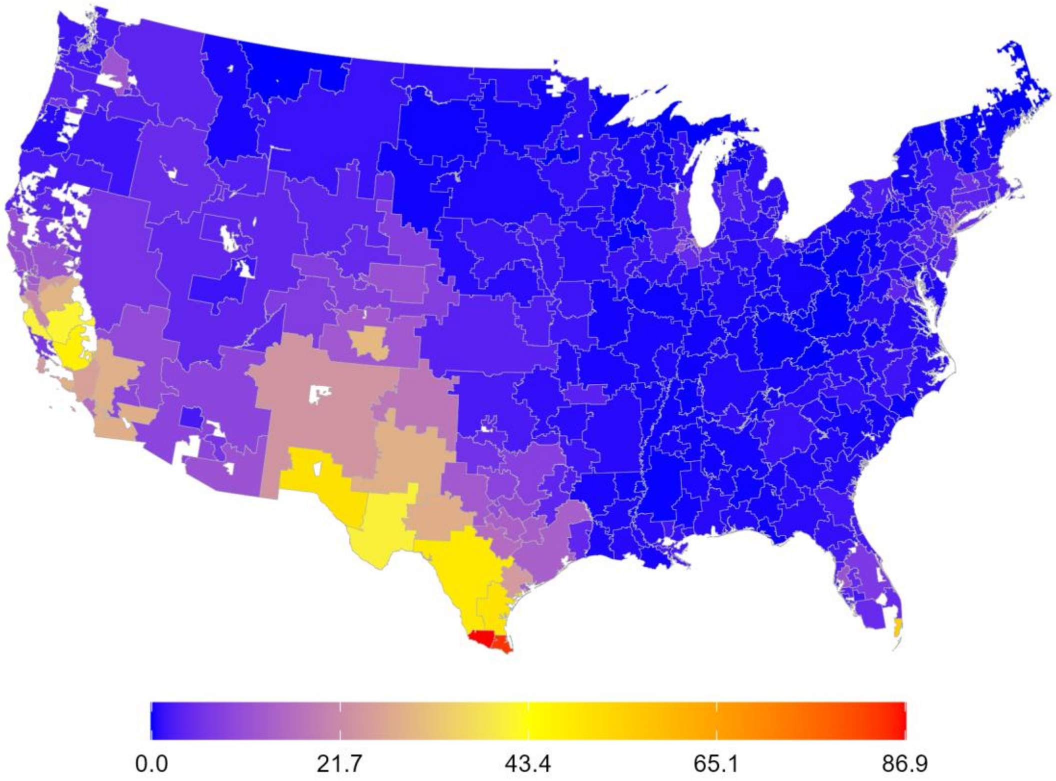

Note: Alaska and Hawaii HRRs are omitted from the map for visualization purposes but are included in the model. The proportions of Hispanic DLE wound patients were 1.8% and 1.7%, respectively.

© 2026 Popescu I et al. *JAMA Network Open*.

eFigure 3. Population-weighted Area Deprivation Index (ADI), averaged for 2017-2019

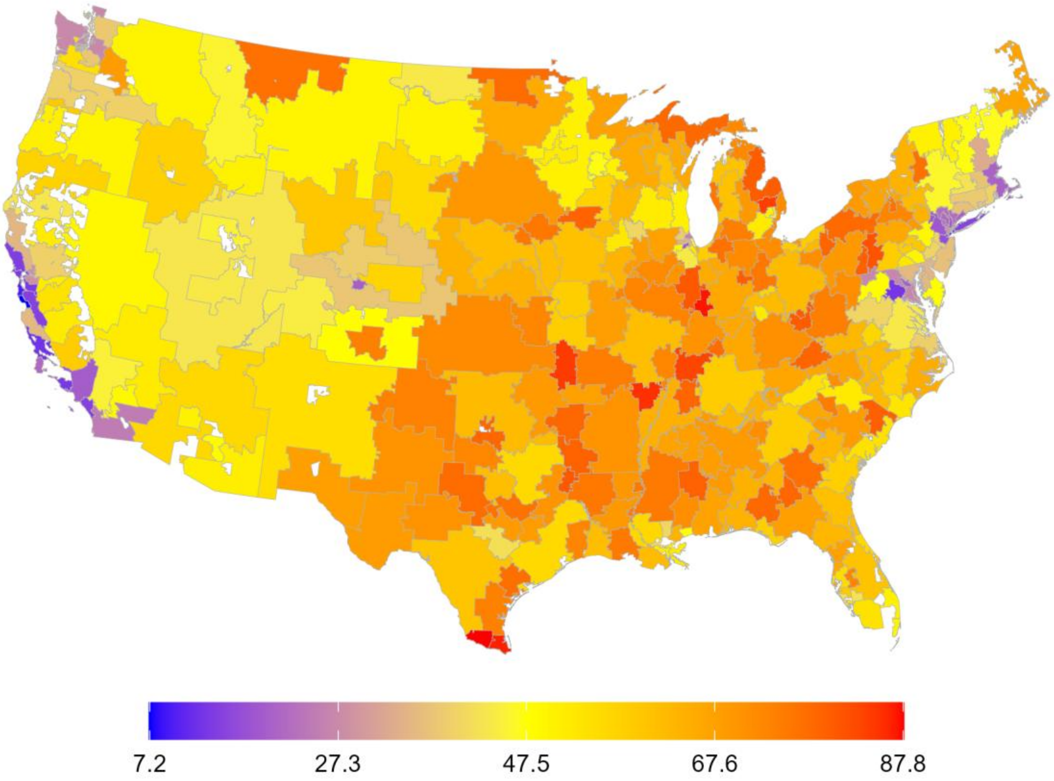

Note: Alaska and Hawaii are omitted from the map for visualization purposes but are included in the model. The weighted ADI for these 2 HRRs was 33.4 and 17.4, respectively.

© 2026 Popescu I et al. *JAMA Network Open*.

eFigure 4. Population-weighted rurality, averaged for 2017-2019

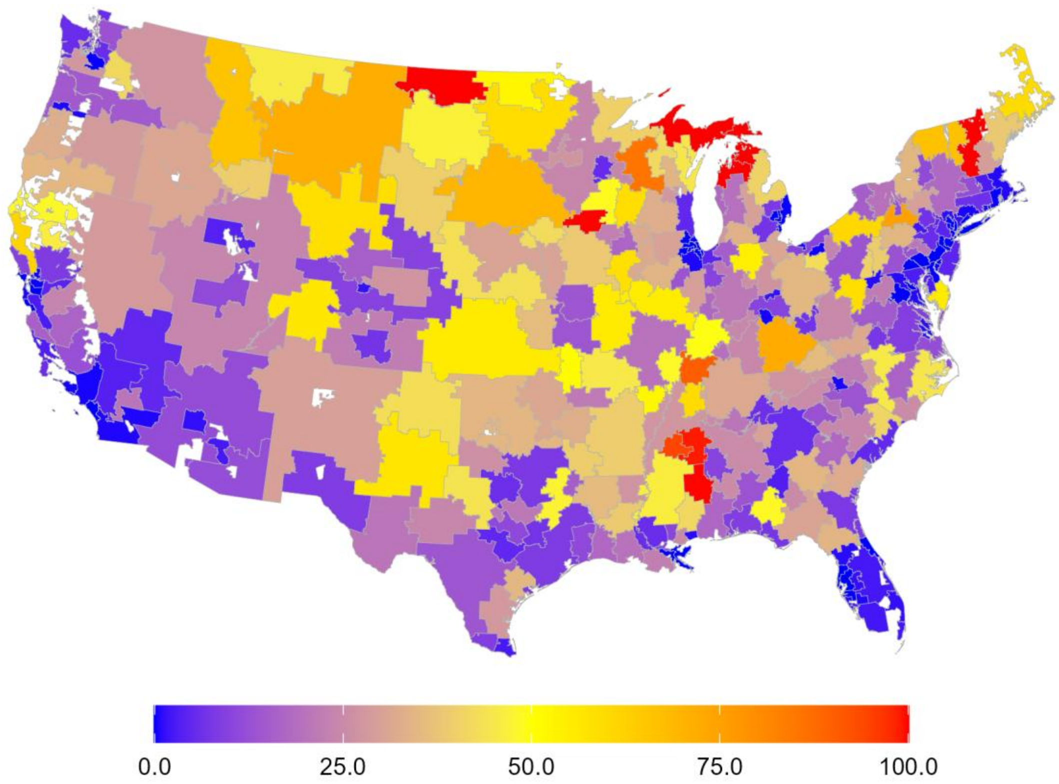

Note: Alaska and Hawaii are omitted from the map for visualization purposes but are included in the model. The weighted rurality for these 2 HRRs was 34.2% and 23.1%, respectively.

© 2026 Popescu I et al. *JAMA Network Open*.

eTable 3. Bayesian multivariable model results: comparing main analysis and sensitivity analysis results using alternative imputation methods for missing AHA survey information on hospital-based wound management programs

	Main results		Sensitivity analysis results	
**HRR Variable**	**OR**	**95% CI***	**OR**	**95% CI***
%Black Medicare DLE wound patients	5.19**	3.78-7.12	5.10**	3.70-7.04
%Hispanic Medicare DLE wound patients	2.39 **	1.73-3.29	2.36**	1.71-3.27
Population weighted ADI	1.29**	1.02-1.62	1.29**	1.02, 1.62
% Rural population	1.12	0.94-1.35	1.14	0.94-1.37
%Hospitals with wound management programs	1.02	0.87-1.18	1.00	0.87-1.16
Primary care physician supply	1.19	0.98-1.45	1.16	0.94-1.44
Podiatrist supply	0.74***	0.66-0.82	0.73***	0.66-0.82
Revascularization-performing specialist supply	1.03	0.87-1.21	1.02	0.86-1.20
Endocrinology supply	0.99	0.92-1.07	0.99	0.92-1.07
Other relevant surgical specialties	1.06	0.90-1.24	1.05	0.90-1.24

*The 95% credible interval (CI) is interpreted similarly to a confidence interval, in that there is a 95% probability that the true OR lies with in the credible interval range.

**the Bayesian posterior probabilities are ≥97.5% indicating a large magnitude positive association

***the Bayesian posterior probability is ≤2.5% indicating a large magnitude negative association

© 2026 Popescu I et al. *JAMA Network Open*.
