# Supplementary material for: Geographic Variation in Amputations for Medicare Patients With Diabetic Lower-Extremity Wounds
Source: JAMA Netw Open. 2026 May 19;9(5):e2613616. doi: 10.1001/jamanetworkopen.2026.13616 (PMC13187873; doi:10.1001/jamanetworkopen.2026.13616)
Supplement: Supplement 2. — Data Sharing Statement [file jamanetwopen-e2613616-s002.pdf]

Data Sharing Statement

Popescu. Geographic Variation in Amputations for Medicare Patients With Diabetic Lower Extremity Wounds. *JAMA Netw Open*. Published May 19, 2026. doi:10.1001/jamanetworkopen.2026.13616

Data
Data available: No

Additional Information
Explanation for why data not available: The study team used Medicare claims and enrollment data that were obtained under a Data Use Agreement with the Centers for Medicare and Medicaid Services (CMS). Researchers interested in accessing the Medicare data need to apply for and obtain approval for data access and enter into a DUA with CMS. A Data Sharing Statement is preovided as a seprate attachment.
